# The Herbal Compound “Diwu Yanggan” Modulates Liver Regeneration by Affecting the Hepatic Stem Cell Microenvironment in 2-Acetylaminofluorene/Partial Hepatectomy Rats

**DOI:** 10.1155/2015/468303

**Published:** 2015-01-05

**Authors:** Bin-Bin Zhao, Han-Min Li, Xiang Gao, Zhi-Hua Ye, Si-Si Cheng

**Affiliations:** ^1^Hubei University of Traditional Chinese Medicine, Wuhan, Hubei 430065, China; ^2^Hepatopathy Institute, Hubei Provincial Hospital of Traditional Chinese Medicine, Wuhan, Hubei 430061, China

## Abstract

*Ethnopharmacological Relevance.* “Diwu Yanggan” (DWYG) has been reported to regulate liver regeneration, modulate the immune response, ameliorate liver injury, kill virus, ameliorate liver fibrosis, and suppress hepatic cancer. However, its mechanisms are still unknown.* Objectives.* To investigate the effects of DWYG on oval cell proliferation in 2-AAF/PH rats and determine its mechanism.* Methods.* Wistar rats were randomly distributed into normal group, sham group, vehicle group, and DWYG group. Hepatic pathological changes were examined by H&E staining. The oval cell markers CD34, AFP, CK-19 and hematopoietic cell markers CD45, Thy1.1, and hepatocyte marker ALB were examined with immunohistochemistry. The percentage of CD34/CD45 double-positive cells in bone marrow was detected by flow cytometry. Cytokine levels were measured with the Bio-plex suspension array system.* Results*. DWYG significantly increased the survival rates of 2-AAF/PH rats and promoted liver regeneration. Furthermore, DWYG increased the ratio of CD34/CD45 double-positive cells on days 10 and 14. In addition, DWYG gradually restored IL-1, GRO/KC, and VEGF levels to those of the normal group.* Conclusions.* DWYG increases 2-AAF/PH rat survival rates, suppresses hepatic precarcinoma changes, and restores hepatic tissue structure and function. DWYG may act by modulating the hepatic microenvironment to support liver regeneration.

## 1. Introduction

Orthotopic liver transplantation is currently the only definitive therapy for patients with end-stage liver diseases; however, severe complications such as acute allograft rejection commonly occur. An ideal treatment would use cells and tissues to supplement organ functionality to avoid transplantation [1–3]. Stem cell and progenitor cell transplantation, including hepatocytes and hepatic oval cells, that have self-renewal and differentiation potential have been utilized as alternative approaches for mediating damaged tissue repair to facilitate liver regeneration [3, 4]. Oval cells are considered adult liver progenitors, as they can differentiate into mature hepatocytes or biliary epithelial cells [5]. Upon inhibition of mature hepatocyte proliferation, oval cells expand and differentiate into mature hepatocytes and biliary epithelial cells to regenerate liver mass after hepatic damage [6]. In the 2-acetylaminofluorene (2-AAF)/partial hepatectomy (PH) model, hepatocyte proliferation is efficiently suppressed, which forces liver regeneration to depend on oval cell proliferation and differentiation [3, 5]. After normal liver mass, any excess oval cells that have failed to differentiate into mature hepatocytes stop replicating and undergo apoptosis to prevent liver hyperplasia [5, 7, 8]. It has been previously reported that hepatic oval cells express the hematopoietic stem cell markers Thy-1 and CD34, which support the hypothesis that hepatic oval cells originate from bone marrow [9–11].

Each organ microenvironment is highly regulated and plays an important role in maintaining stem cell division by modulating signaling [6]. The microenvironment comprises the extracellular matrix, epithelial and nonepithelial resident liver cells, and recruited inflammatory cells, as well as a variety of growth-modulating molecules [3, 6]. Cytokines are important for the balance of microenvironment and play an important role in oval cell-dependent liver regeneration [12, 13].

The Chinese herbal medicine formula Diwu Yanggan (DWYG) is a new drug authorized by the Hubei Food and Drug Administration (Grant no. Z20113160). The invention patent number from the People's Republic of China State Intellectual Property Office is 201210580999.2. The mixture includes five Chinese medicinal herbal extracts, whose proportions (w/w) are as follows:* Rehmannia glutinosa* (Gaertn.) DC., 20.0%;* Artemisia scoparia* Waldst. & Kitam., 33.3%;* Curcuma longa* L., 13.4%;* Schisandra chinensis *(Turcz.) Baill., 20.0%; and* Glycyrrhiza uralensis* Fisch., 13.4%.

DWYG has been reported to regulate liver regeneration, modulate the immune response, ameliorate liver injury, kill virus, ameliorate liver fibrosis, and suppress hepatic cancer [14, 15]. Although several studies have focused on hepatic oval cell regulation, efficient strategies to modulate oval cell-mediated liver regeneration do not yet exist. In the present study, we demonstrate that DWYG modulates liver regeneration in 2-AAF/PH rats, potentially through the modulation and restoration of the liver regeneration microenvironment.

## 2. Materials and Methods

### 2.1. Materials

Rat lymphocyte separation buffer was purchased from Hao Yang Biological Manufacture Co., Ltd (Tianjin, China). PE-anti-CD34, FITC-anti-CD45, PE-anti-rat IgG, anti-CD45, anti-ALB, anti-AFP, anti-CK19, anti-Thy1.1, FITC-anti-rat IgG, and PE-anti-CD34 antibodies were purchased from Santa Cruz Biotechnology (Santa Cruz, CA, USA). 2-Acetylaminofluorene was purchased from Sigma Aldrich (Saint Louis, MO, USA). The SABC Immunohistochemistry Kit was purchased from Boster Biotechnology (Wuhan, China), and the Bio-plex suspension array system was purchased from Bio-Rad Laboratories (Hercules, CA, USA).

The DWYG capsules used in this study (batch number 20120221) were provided by the Traditional Chinese Medicine Preparation Room of Hubei Provincial Hospital of Traditional Chinese Medicine. Briefly, the DWYG capsules were prepared as follows: the* Rehmannia glutinosa* (Gaertn.) DC. and* Glycyrrhiza uralensis* Fisch. decoction and the* Schisandra chinensis* (Turcz.) Baill.,* Artemisia scoparia* Waldst. & Kitam., and* Curcuma longa* L. coarse powders were mixed together, and 75% ethanol was added and extracted by reflux extraction three times. The ethanol extracts were filtrated, concentrated, and recovered by reduced pressure. The DWYG capsules were obtained by decompression drying and granulating of the above-refined concentrates. DWYG capsules were suspended in distilled water at a final concentration of 36 mg/mL. Two representative components of herbal medicines were examined by high-performance liquid chromatography (HPLC) for quality control.

### 2.2. Animal Model

Male Wistar rats (180 ± 10 g) were purchased from Hubei Province Experimental Animal Research Center. The rats were fed pellet food and water* ad libitum *in plastic cages at 21 ± 2°C and kept on a 12-hour light-dark cycle. Animal welfare and experimental procedures were carried out in strict accordance with the Guide for the Care and Use of Laboratory Animals (The Ministry of Science and Technology of China, 2006) and the related ethical regulations of our hospital. All efforts were made to minimize animals' suffering and to reduce the number of animals used.

The 2-AAF/PH model was modified from previous reports [5]. Briefly, the rats were distributed into four groups: normal, sham, vehicle, and DWYG. For the sham group, rats' abdomens were opened, and 1 mL whole blood was taken from the portal vein. No partial hepatectomy (PH) was performed. For vehicle and DWYG groups, the rats received PH. The three groups were then orally administered 20 mg/kg 2-AAF once a day for one week. Meanwhile, the sham and vehicle groups were orally administered distilled water (10 mL/kg) until sacrificed, while the rats of the DWYG group were orally administrated 10 mL/kg DWYG until sacrificed. Sixteen rats in each group were sacrificed on days 8, 10, 14, 17, 19, and 22 after PH. Liver sections and bone marrow were collected. The survival rate was recorded and analyzed.

### 2.3. Hematoxylin-Eosin Staining and Immunohistochemical Staining

For hematoxylin-eosin (H&E) staining, liver paraffin sections were dewaxed and rehydrated, and the sections were stained with H&E. Histological observations were made by light microscopy. For immunohistochemical staining, paraffin sections were dewaxed and hydrated and stained with the indicated primary antibodies. The coloration reaction was performed according to the SABC Kit (SolarBio, Beijing, China) manufacturer's protocols. Images were acquired with a Nikon TE2000-U microscope (Nikon, Kyoto, Japan), and the mean integral optical density (IOD) was analyzed with Image Pro-Plus 6.0 software (Media Cybernetics, Rockville, MD, USA).

### 2.4. Flow Cytometry Analysis

Flow cytometry was performed as previously described [10]. Briefly, bone marrow cells were separated from thigh femurs and stained with PE-anti-CD34 and FITC-anti-CD45 antibodies at room temperature for 30 minutes, and the cells were washed twice with saline and analyzed by FACSCalibur (BD Biosciences, San Jose, CA, USA). The percentage of CD34/CD45 double-positive cells was calculated and analyzed.

### 2.5. Cytokine Detection

Serum was obtained upon sacrifice, and cytokine levels were detected by Bio-plex suspension array system (Bio-Rad Laboratories, Berkeley, CA, USA) and analyzed with SPSS 19.0 software (IBM, Armonk, NY, USA).

### 2.6. Statistical Analysis

Data are shown as means ± standard deviation (SD). Data analysis was performed by SPSS 19.0 software. A one-way ANOVA test was performed to compare the differences between more than two groups. A value of *P* < 0.05 was considered to be statistically significant. Survival rates were recorded and analyzed by Log-rank (Mantel-Cox) test. A value of *P* < 0.05 was considered to be statistically significant.

## 3. Results

### 3.1. DWYG Significantly Increases 2-AAF/PH Rat Survival Rates

**Figure 1:**
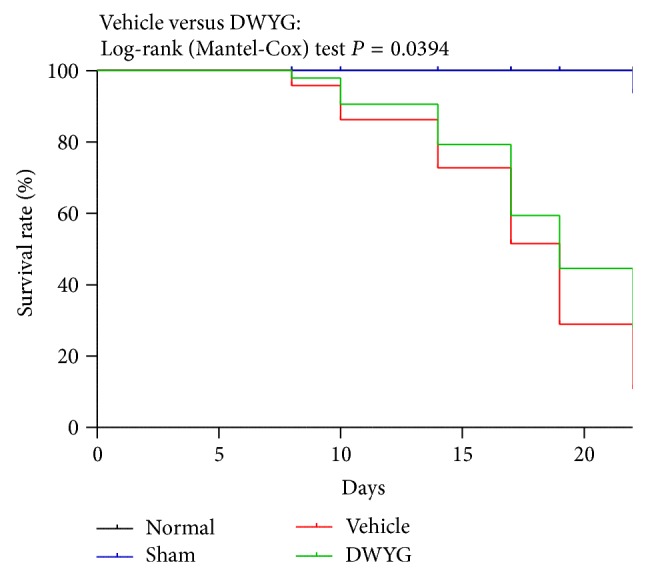
DWYG influences the survival rate of 2-AAF/PH rats. Normal Wistar rats (normal group), sham-operated rats (sham group), and 2-AAF/PH rats were orally administered vehicle (distilled water, vehicle group) or DWYG (DWYG group, 10 mg/kg). Survival rates were recorded and analyzed by Log-rank (Mantel-Cox) test.

Normal Wistar rats (normal group), sham-operated rats (sham group), and 2-AAF/PH rats were orally administered vehicle (distilled water, vehicle group) or DWYG (DWYG group, 10 mL/kg). Survival rates were recorded and analyzed by Log-rank (Mantel-Cox) test. Compared to the vehicle group, DWYG significantly (*P* = 0.0394, *n* = 96) increased 2-AAF/PH rat survival rates (Figure 1).

### 3.2. DWYG Promotes Liver Regeneration in 2-AAF/PH Rats

**Figure 2:**
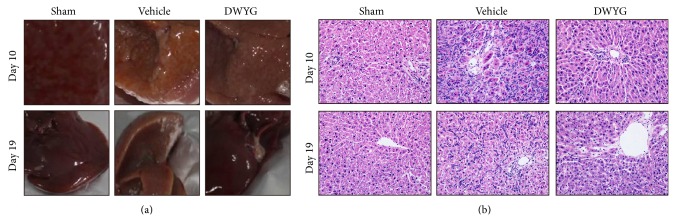
DWYG affects hepatic oval cell proliferation in 2-AAF/PH rats. Normal Wistar rats (normal group), sham-operated rats (sham group), and 2-AAF/PH rats were orally administered vehicle (distilled water, vehicle group) or DWYG (DWYG group, 10 mg/kg). Paraffin liver sections were dewaxed and rehydrated, and the sections were stained with H&E. (a) Photos of representative liver. (b) Representative images of H&E staining. Original magnification, 200x.

We treated rats as described above and sacrificed them on days 8, 10, 14, 17, 19, and 22 and collected their livers. As shown in Figure 2(a), we observed tuberculous pathological changes in livers of the sham and vehicle groups. Compared to the vehicle group, DWYG-treated livers exhibited significantly healthier-looking livers. H&E staining revealed that hepatic cords of 2-AAF/PH rats were destroyed and small neoformatively basophilia liver cell nodes had formed. Moreover, there were increased bile canaliculi and central veins, and oval cells formed abundant long-cable-like tubular structures. On day 19, vehicle group livers exhibited increased proliferative mitotic oval cells. Strikingly, DWYG ameliorated the above pathological changes and structural disorganization (Figure 2(b)).

### 3.3. DWYG Dynamically Affects 2-AAF/PH Liver Immunohistochemical Indices

**Figure 3:**
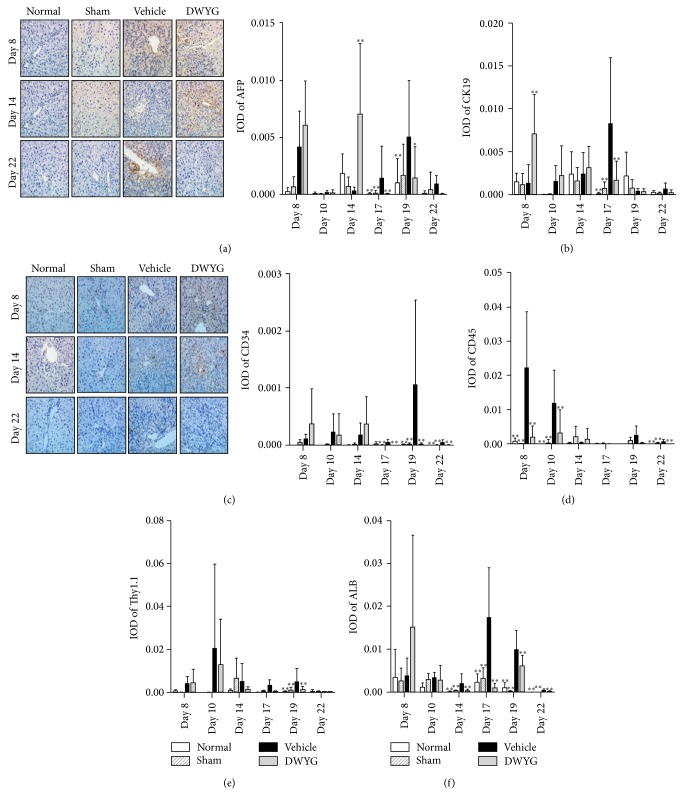
Effects of DWYG on 2-AAF/PH rat liver immunohistochemical indices. Normal Wistar rats (normal group), sham-operated rats (sham group), and 2-AAF/PH rats were orally administered vehicle (distilled water, vehicle group) or DWYG (DWYG group, 10 mg/kg). Paraffin sections were dewaxed and rehydrated and stained with the indicated primary antibodies. The mean integral optical density (IOD) was analyzed with Image Pro-Plus 6.0 software. (a) Representative images (left panel) of AFP immunohistochemistry and IOD (right panel). (b) IOD of CK19. (c) Representative images (left panel) of CD34 immunohistochemistry and IOD (right panel). (d–f) IOD of CD45 (d), Thy1.1, (e) and ALB (f). Data shown are means ± SD of 16 rats in each group. ^*^ *P* < 0.05, ^**^ *P* < 0.01 versus vehicle group. Original magnification, 200x.

In the following experiments, we investigated changes in immunohistochemical indices in 2-AAF/PH livers treated with vehicle or DWYG. We examined protein levels of the oval cell markers AFP, CD34, and CK19, the hepatocyte marker ALB, and the hematopoietic cell markers CD45 and Thy1.1 by immunohistochemistry and analyzed their mean integral optical density (IOD) (Figure 3). DWYG significantly and transiently increased the IOD of the oval cell marker AFP on day 14 (0.00704 ± 0.00621 versus 0.00030 ± 0.00032, *P* < 0.01), which then decreased on day 17 (0.00001 ± 0.00003 versus 0.0014 ± 0.0028, *P* < 0.01) and day 19 (0.00144 ± 0.00273 versus 0.00503 ± 0.00494, *P* < 0.05), compared to the vehicle group (Figure 3(a)). Similarly, DWYG significantly increased CK-19 IOD on day 8 (0.00714 ± 0.00457 versus 0.00138 ± 0.00215, *P* < 0.01), which then decreased on day 17 (0.00169 ± 0.00229 versus 0.00836 ± 0.00770, *P* < 0.01), compared to the vehicle group (Figure 3(b)). However, DWYG significantly decreased a third oval cell marker CD34 on day 17 (0.00000 ± 0.00000 versus 0.00004 ± 0.00006, *P* < 0.01), day 19 (0.00001 ± 0.00002 versus 0.00106 ± 0.00149, *P* < 0.01), and day 22 (0.00000 ± 0.00000 versus 0.00004 ± 0.00004, *P* < 0.01, Figure 3(c)). DWYG significantly decreased the hematopoietic cell marker CD45 IOD on day 8 (0.00182 ± 0.00336 versus 0.02223 ± 0.01637, *P* < 0.01), day 10 (0.00311 ± 0.00680 versus 0.01182 ± 0.00432, *P* < 0.01), and day 22 (0.00000 ± 0.00000 versus 0.00062 ± 0.00056, *P* < 0.01, Figure 3(d)). It also significantly decreased Thy1.1 IOD on day 19 (0.00116 ± 0.00154 versus 0.00488 ± 0.00628, *P* < 0.01, Figure 3(e)). As shown in Figure 3(f), DWYG significantly decreased the hepatocyte marker ALB IOD on day 14 (0.00030 ± 0.00039 versus 0.00193 ± 0.00228, *P* < 0.01), day 17 (0.00092 ± 0.00106 versus 0.01740 ± 0.01170, *P* < 0.01), day 19 (0.00602 ± 0.00253 versus 0.00989 ± 0.00447, *P* < 0.01), and day 22 (0.00009 ± 0.00017 versus 0.00044 ± 0.00019, *P* < 0.01). These results suggest that DWYG has distinct influences on liver immunohistochemical indices at different time points after 2-AAF/PH induction.

### 3.4. DWYG Influences Myeloid Cell Differentiation in 2-AAF/PH Rats

**Figure 4:**
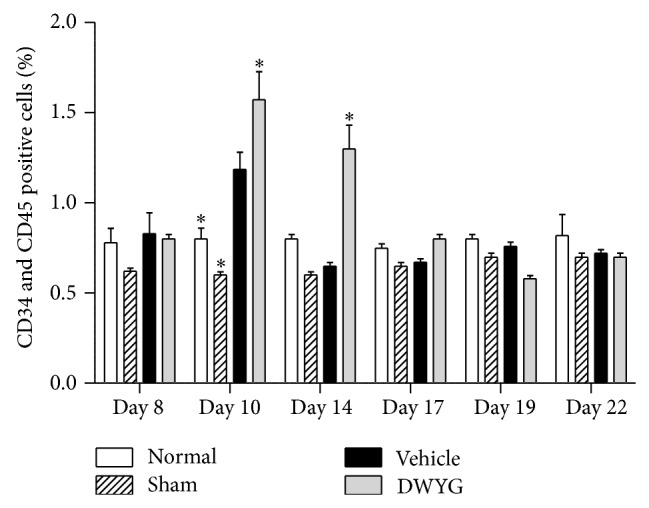
Effects of DWYG on the percentage of CD34/CD45 double-positive myeloid cells in 2-AAF/PH rats. Normal Wistar rats (normal group), sham-operated rats (sham group), and 2-AAF/PH rats were orally administered vehicle (distilled water, vehicle group) or DWYG (DWYG group, 10 mg/kg). Bone marrow cells were separated, and the percentage of CD34/CD45 double-positive cells was analyzed by flow cytometry. Data shown are means ± SEM of 16 rats in each group. ^*^ *P* < 0.05 versus vehicle group.

Because we saw a decrease in differentiated cell marker expression and a transient expression in progenitor oval cell markers, we aimed to determine changes in each population. We next examined the ratio of CD34 and CD45 double-positive cells in the bone marrow of 2-AAF/PH rat. As shown in Figure 4, the percentage of CD34/CD45 double-positive cells significantly increased (*P* < 0.05) in the vehicle group (1.36 ± 0.10) compared to normal (0.80 ± 0.10) and sham groups (0.54 ± 0.03). DWYG significantly (*P* < 0.05) increased the percentage of CD34/CD45 double-positive cells on day 10 and day 14. These results suggest that DWYG promotes the differentiation of bone marrow cells in 2-AAF/PH rats.

### 3.5. DWYG Affects Cytokine Levels in 2-AAF/PH Rats

**Figure 5:**
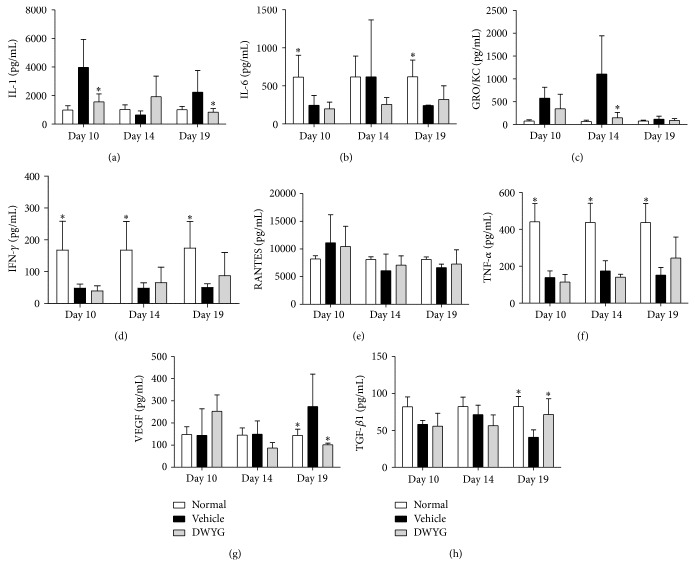
Effects of DWYG on cytokine levels in 2-AAF/PH rats. Normal Wistar rats (normal group) and 2-AAF/PH rats were orally administered vehicle (distilled water, vehicle group) or DWYG (DWYG group, 10 mg/kg). Serum was obtained when the rats were sacrificed, and cytokine levels were detected by Bio-plex suspension array system. (a–h) Serum levels of IL-1 (a), IL-6 (b), GRO/KC (c), IFN-γ (d), RANTES (e), TNF-α (f), VEGF (g), and TGF-β1 (h). Data shown are means ± SD of 16 rats in each group. ^*^ *P* < 0.05 versus vehicle group.

In the following experiments, we detected changes in cytokine levels at different time points after 2-AAF/PH induction. As shown in Figure 5, levels of IL-1 (Figure 5(a)), GRO/KC (Figure 5(c)), and VEGF (Figure 5(g)) were significantly (*P* < 0.05) higher in the vehicle group compared to the normal group, while the levels of IL-6 (Figure 5(b)), IFN-*γ* (Figure 5(d)), TNF-*α* (Figure 5(f)), and TGF-*β*1 (Figure 5(h)) were significantly lower in the vehicle group compared to the normal group (*P* < 0.05). Compared to the vehicle group, DWYG rescued cytokine levels more closely to those in the normal group, and the levels of IL-1 (Figure 5(a)), GRO/KC (Figure 5(c)), and VEGF (Figure 5(g)) gradually returned to normal levels. These results imply that DWYG treatment allows the recovery of cytokine levels to normal levels, allowing for liver regeneration after 2-AAF/PH treatment.

## 4. Discussion

Liver regeneration is thought to be achieved by hepatic cells (the most efficient liver stem cells), oval cells (intraliver stem cells), and bone marrow derived stem cells [7, 11, 16]. In the 2-AAF/PH model, hepatocyte proliferation is efficiently suppressed by 2-AAF, which forces liver regeneration to become dependent on oval cell replication and differentiation [3, 5]. Their proliferation and differentiation are determined by the hepatic stem cell microenvironment. A disruption in the microenvironment leads to disordered liver regeneration. By ameliorating the hepatic stem cell microenvironment, liver regeneration can be efficiently regulated. In this study, we found that 2-AAF suppressed liver regeneration because it was toxic to the liver, resulting in a high death rate of 2-AAF/PH rats. The death rate of rats in the vehicle group was significantly higher compared to that of normal and sham groups. DWYG significantly rescued the death rate of 2-AAF/PH rats and improved pathological liver damage in 2-AAF/PH rats, with a more complete recovery.

In 2-AAF/PH rats, the major hepatic stem cells participating in liver regeneration were hepatic oval cells and bone marrow stem cells. Petersen et al. have previously reported that oval cells or other hepatic cells originate from the bone marrow [11]. Reports by Oh et al. also support the bone marrow origination of hepatic oval cells [17]. Hepatic oval cells are the seed of liver regeneration but can also be hepatic carcinoma (HCC) progenitor cells, suggesting that oval cells are characteristic hepatic stem cells during liver regeneration and cancer initiation. Disruption of the microenvironment is an important factor for oval cell transition into HCC stem cells. 2-AAF/PH is a hepatic precancerous model, and we found that the precancerous lesions in the vehicle group were more severe than those in the normal and sham groups. DWYG bidirectionally regulated oval cell proliferation and differentiation, as it promoted bone marrow stem cell and oval cell proliferation in early stages (8–14 days after PH) to support liver regeneration; it also suppressed excessive oval cell proliferation in later stages (17–22 days after PH) and abnormal differentiation to prevent cancerous hepatic cell lesions.

DWYG increased the percentage of CD34/CD45 double-positive cells in 2-AAF/PH rats, which may be associated with DWYG's promotion of bone marrow hematopoietic cell proliferation, accelerating oval cell generation to achieve liver regeneration. We did not observe excessive bone marrow hematopoietic cell proliferation upon the completion of liver regeneration. Combined with the increased CD34 protein levels, we hypothesize that the promotion of bone marrow cells proliferation would increase the percentage of CD34-positive oval cells to accelerate liver regeneration. Thy1.1 and CD45 are hematopoietic stem cell markers. Compared to vehicle, DWYG significantly decreased Thy1.1 and CD45 protein levels and accelerated peak ALB expression. These results suggest that DWYG efficiently promotes oval cell differentiation into bile duct cells and hepatocytes to facilitate liver regeneration.

In conclusion, our results suggest that DWYG increases 2-AAF/PH rat survival, suppresses hepatic precarcinoma changes, and restores hepatic tissue structure and function. We suggest that DWYG acts by promoting bone marrow stem cell and oval cell proliferation during early stages (8–14 days after PH) to facilitate liver regeneration, while suppressing bone marrow stem cell differentiation to hepatic oval cells during later stages (17–22 days after PH) to prevent hepatic carcinogenesis. DWYG maymodulate liver regeneration by modulating the hepatic stem cell microenvironment, controlling oval cell proliferation, and bone marrow stem cell differentiation.

## Abbreviations

AFP: Alpha-fetoprotein
CK-19: Cytokeratin-19
Thy1.1: Thymus cell antigen 1, theta
ALB: Albumin
2-AAF/PH: 2-Acetylaminofluorene/partial hepatectomy
HPLC: High-performance liquid chromatography
H&E: Hematoxylin-eosin
SD: Standard deviation
IOD: Integral optical density
IL-1: Interleukin-1
GRO/KC: Growth-related oncogene, systemic name: CXCL1
IL-6: Interleukin-6
VEGF: Vascular endothelial growth factor
TNF-*α*: Tumor necrosis factor alpha
TGF-*β*1: Transforming growth factor-*β*1
IFN-*γ*: Interferon-gamma
HCC: Hepatic carcinoma.
